# Stage III Colon Cancer: The Individualized Strategy of Adjuvant Chemotherapy for Aged Under and Over 70

**DOI:** 10.1371/journal.pone.0138632

**Published:** 2015-09-18

**Authors:** Chieh-Sheng Lu, Ping-Ying Chang, Yu-Guang Chen, Jia-Hong Chen, Yi-Ying Wu, Ching-Liang Ho

**Affiliations:** 1 Department of Internal Medicine, Kaohsiung Armed Forces General Hospital, Kaohsiung, Taiwan; 2 Division of Hematology and Oncology, Department of Internal Medicine, Tri-Service General Hospital, National Defense Medical Center, Taipei, Taiwan; Shanghai Jiao Tong University School of Medicine, CHINA

## Abstract

**Background:**

The aim of this study was to examine the specific chemoregimens selected for adjuvant therapy in the patients with stage III colon cancer. We investigated the trends in chemotherapeutic prescribing patterns and looked for adequate therapeutic setting for these patients.

**Methods:**

288 patients presenting with stage III colon cancer and undergoing adjuvant therapies after curative surgery for more than 3-month were enrolled between January 2006 and December 2011. Demographic characteristics and therapeutic factors were analyzed, including age, gender, histological grade, tumor sizes, tumor location, pathologic stage, performance status, serum carcinoembryonic antigen, regimens selection, interval from the operation to the start of adjuvant therapy and prolonged adjuvant therapy. Kaplan– Meier methods were utilized for drawing survival curves and Cox model was used to analyze survival, prognostic factors.

**Results:**

The analysis showed that the patients aged under 70 received more intensive therapies than those aged over 70 (*P*<0.001). Later, advanced analysis in therapeutic factors was conducted between the patients aged under 70 and those over 70. In the patients aged under 70, significant differences in 4-year overall survival (OS) were noted between UFUR (oral tegafur-uracil plus leucovorin) groups and FOLFOX (5-FU plus oxaliplatin) [65.6% versus (vs) 89.8%, relative risk (RR) 3.780, 95% confidence interval (CI) 1.263–11.315, *P* = 0.017]. There were also differences in 4-year OS between these patients with and without oxaliplatin-contained regimens (92.1% vs 83.4%, respectively, RR 0.385, 95% CI 0.157–0.946, *P* = 0.037). In addition, the patients who received intravenous or combined therapy also had higher 4-year OS than those only received oral regimens (92.1% vs 76.6%, *P* = 0.077), though the finding did not reach statistical significance. In contrast to the survival benefits of above therapeutic settings for the patients aged under 70, there was less advantage in the old patients when they received intensive therapies or even oxaliplatin-contained regimens. Prolonged cycles of adjuvant therapy resulted in no significant benefit to survival rates regardless of ages.

**Conclusions:**

The adequate individualized therapeutic strategy plays an important role for stage III colon cancer. Our findings suggested that benefit of oxaliplatin-contained therapy is limited to patients aged under 70 and oral fluoropyrimidines may be an effective option for old patients. In addition, prolonged adjuvant setting is suggested to be unbeneficial for managing stage III colon cancer.

## Introduction

Colon cancer is one of the most common cancers worldwide. In 2011, 7507 persons were diagnosed with this cancer in Taiwan and 2003(26.7%) patients were confirmed as stage III disease [1]. Efficacy of adjuvant chemotherapy after primary surgery for stage III colon cancer has been evaluated [2–7]. Despite the therapeutic benefit from adjuvant therapy, use of adjuvant chemotherapy varies among patients in Taiwan, especially in elderly patients due to a consideration of therapeutic convenience or cost. Limited studies, however, have examined the specific chemotherapy regimens which are developed based on type of adjuvant therapy, therapy duration, or individual characteristics associated [8].

In the present study, we analyzed the clinical data of the patients with stage III colon cancer, who received primary surgery followed by adjuvant chemotherapy at Tri-Service General Hospital. Based on the medical records, we were able to elucidate the trends in prescribing pattern of chemotherapeutic agents and to explore the association between patients’ characteristics and decision making for prescription.

## Material and Methods

### Ethic statement

This retrospective study was conducted based on population-based data from the Cancer Registry Group, Tri-Service General Hospital, Taipei, Taiwan and under the guidelines of the Helsinki Declaration, approved by the Human Subjects Protection Offices (IRB) at the Tri-Service General Hospital (TSGHIRB number: 2-104-05-021). Because all identifying patient information was removed prior to analysis in this study, informed consent was not obtained.

### Patient selection

This was a observational study. A total of 316 patients with newly diagnosed stage III colon cancer who received adjuvant chemotherapy following curative surgery were identified between January 2006 and December 2011. The database contained detailed information about patient characteristics, operative findings, histological examination, laboratory findings, and adjuvant therapies. The follow-up survival data were collected retrospectively based on medical record analyses. Cancer was staged using the American Joint Committee on Cancer (AJCC) 7th edition TNM [the extent of the tumor (T), the extent of spread to the lymph nodes (N), and the presence of metastasis (M)] classification: colon [9]. Patients who were with 2 or more cancers, other types of cancers preoperatively, without clear information about the chemotherapy (regimens or cycles) or received adjuvant chemotherapy less than 3 months were excluded. Analysis was carried out on the data of 288 patients.

### Chemotherapy adjuvant regimens

All 288 patients started receiving adjuvant chemotherapy after curative surgery. In Taiwan, the National Health Insurance (NHI) supported the oxaliplatin-contained regimens as adjuvant therapy for the patients with stage III colon cancer after 2009. During this period, however, given the expected benefits and possible risks of toxicity, a consensus has not been reached as to whether adjuvant regimens containing oxaliplatin should be given to these patients. Due to the availability and convenience of oral 5-FU derivatives such as oral tegafur-uracil or capecitabine, patients received sundry therapeutic strategies: (1) FOLFOX: Oxaliplatin 85 mg/m^2^ intravenous (IV) over 2 hours, leucovorin 400 mg/m^2^ IV over 2 hours, 5-Fluorouracil (5-FU) 400 mg/m^2^ IV bolus on day 1 and then 5-FU 1200 mg/m^2^/day continuous infusion for 2 days (total 2400 mg/m^2^ over 46–48 hours), repeated every 2 weeks for total 12 cycles [6,10,11]. (2) XELOX- CapeOx: Oxaliplatin 130 mg/m^2^ over 2 hours on day 1 and capecitabine 1000 mg/m^2^ twice daily from days 1 to 14, every 3 weeks as one cycle, for total 24 weeks [12,13]. (3) FL- 5-Fluorouracil (5-FU)/leucovorin: Leucovorin 500 mg/m^2^ given as a 2-hour infusion, 5-FU 500 mg/m^2^ given bolus 1 hour after the start of leucovorin, and repeated weekly for 24 cycles [14] or leucovorin 400 mg/m^2^ intravenous (IV) over 2, hours on day 1, followed by 5-FU bolus 400 mg/m^2^ and then 1200 mg/m^2^/day continuous infusion for 2 days (total 2400mg/m^2^ over 46–48 hours), repeat every 2 weeks for 12 cycles [15]. (4) XELODA- Oral capecitabine: Capecitabine 1250 mg/m^2^ twice daily from day 1 to 14, every 3 weeks as one cycle, for total 8 cycles [4]. (5) UFUR- Oral UFUR/LV: tegafur-uracil 300 mg/m^2^ daily plus oral folinate calcium (leucovorin) 30mg every 8 hours for 28 days with a subsequent pause of 7 days, every 5 weeks as one cycle, for 5 cycles [16–18]. (6) OXA-based+ 5-FU-based: modified oxaliplatin-based regimens, such as FOLFOX or XELOX, for more than 6 cycles followed by only 5-FU-based regimens, such as FL, oral XELODA or UFUR, for total 6-month treatment.

### Follow-up

During this cohort study, all patients underwent regular follow-up examinations, including serial serum carcinoembryonic antigen (CEA) measurements every 3 months for at least 3 years (later every 4–6 months), and abdominal sonography or computed tomography (CT) scan, chest X-ray, and colonoscopy every 12 months. However, image surveys were conducted immediately for patients with suspecious disease progression or relapse.

### Statistical Analysis

Patients’ characteristics included sex, age, histological grade, tumor sizes [19], tumor location [20], pathologic stage, Eastern Cooperative Oncology Group (ECOG) performance status (PS), CEA before operation, and positive lymph node ratio (LNR) [21]. The chemotherapy-related parameters were also examined, included regimens selection, postoperative time interval from the operation to the start of adjuvant therapy [22] and adding cycles of adjuvant therapy.

The distribution of baseline patient and characteristics across specific treatment regimens was evaluated using Pearson’s *X* ^2^ test for categorical variables. Kaplan–Meier methods were applied in drawing survival curves and Cox model was used to analyze survival, prognostic factors. The progression-free duration was defined as between the curative surgical day and the day of disease progression, relapse, death or the last day the patient was known to be alive; the duration of overall survival (OS) was defined as the time interval between the diagnostic day and the day of death from any cause or the last day the patient was known to be alive. All P values were two-sided; *P* values less than 0.05 was considered as statistically significant. Data were analyzed using the statistical Package for the Social Sciences (SPSS, IBM PASW Statistics 18, ver 18.0.0, WinWrap Basic, copyright 1993–2007 Polar Engineering and Consulting).

## Results

### Patient cohorts and characteristics

The descriptive characteristics and distribution of patients according to chemoregimens are shown in Table 1. The median observation period (from the day of diagnosis to the final date) for entire study population was 43.5 months. The median age of diagnosis was 64.0 years (range 31–88), and 108 (37.5%) patients were ≧70 years old at diagnosis. Of total 288 patients, 154 (53.5%) were males and 134 (46.5%) were females. Main tumor size over 6 cm was recorded in 59 (20.5%) patients and 116 (40.3%) patients were identified as right colon cancer by imaging. For histological grading of tumor, there were 5 (1.7%) patients with well-differentiation, 247 (85.8%) with moderately-differentiation and 34 (11.8%) with poorly-differentiation. The distributions of patients using TNM staging system were stage IIIA, 26(9.0%); stage IIIB, 188 (65.3%) and stage IIIC, 74 (25.7%). Serum CEA level before operation over 5.00 ng/ml (normal range ≦5.00 ng/ml) was detected in 74 (25.7%) patients and poor performance status (ECOG>2) was identified in 29 (10.1%) patients. There were 126 (43.8%) patients recorded with LNR ≧0.18. The time interval, from curative surgery to the start of adjuvant therapy, more than 2 months was recorded in 24 (8.3%) patients. The duration of chemotherapy varied among different therapeutic strategies and additional therapies more than 2 months were noted in 73 (25.3%) patients. Of 241 patients alive, 47 (19.5%) had disease progression or relapse.

**Table 1:** The descriptive characteristics and distribution of patients according to chemoregimens. Abbreviations: CEA: carcinoembryonic antigen; ECOG: Eastern Cooperative Oncology Group performance status; FL: 5-Fluorouracil (5-FU)/leucovorin; FOLFOX: mFOLFOX 6, 5-FU/leucovorin/oxaliplatin; LNR: the positive lymph node ratio of dissected lymph nodes; No.: number; OXA-based+ 5-FU-based: Oxaliplatin-based chemotherapy (less than 8 cycles of 5-FU/leucovorin/oxaliplatin or less than 6 cycles of oral capecitabine/intravenous oxaliplatin) followed by 5-FU-based chemotherapy (5-FU/leucovorin, oral tegafur-uracil/folinate calcium or oral capecitabine); *P*: probability value; TNM stage: Cancer staging system which was developed and is maintained by the American Joint Committee on Cancer (AJCC) and the Union for International Cancer Control (UICC) is based on the extent of the tumor (T), the extent of spread to the lymph nodes (N), and the presence of metastasis (M); UFUR: oral UFUR/LV, tegafur-uracil/oral folinate calcium (leucovorin); w/o: without; XELODA: oral capecitabine; XELOX: CapeOx, oral capecitabine/intravenous oxaliplatin; y/o: years old.

Patients’ characteristics	No. of total patients	FOLFOX	XELOX	OXA-based+ 5-FU-based	FL	XELODA	UFUR	*P*
**No. of patients**	288	141	23	40	17	18	49	
**Age (y/o)**								
Range	31–88	31–82	49–85	35–88	31–75	42–84	41–88	
Median	64.0	60.0	64.0	63.0	63.0	74.5	78.0	
Mean	64.1±13.0	59.8±12.5	67.7±10.4	62.6±11.8	60.4±11.5	72.2±10.7	74.4±10.7	
≧70	108	38	8	10	4	11	37	**<.001**
<70	180	103	15	30	13	7	12	
**Sex**								
Male	154	81	11	16	5	9	32	.053
Female	134	60	12	24	12	9	17	
**Tumor Site**								
R-colon	116	58	9	14	8	10	17	.665
L-colon	172	83	14	26	9	8	32	
**Pre-operative CEA (ng/ml)**								
Range	0.51–214.00	0.52–200.00	0.74–22.96	1.21–214.00	1.00–5.00	0.54–18.16	0.51–100.00	
Median	2.55	2.32	3.23	2.69	2.55	2.31	3.21	
Mean	8.95	7.46	6.40	20.22	3.14	3.36	9.28	
>5.00	74	33	8	16	0	2	15	**.015**
≦5.00	214	108	15	24	17	16	34	
**Histological Grade**								
Well	5	2	2	0	0	0	1	.085
Moderate	247	114	19	38	16	17	43	
Poor	34	24	2	1	1	1	5	
Data missing	2	1	0	1	0	0	0	
**Tumor Size (cm)**								
Range	0.4–13.0	1.0–13.0	1.2–8.0	0.4–9.0	2.3–9.0	2.5–8.0	1.3–10.0	
Median	4.3	4.0	3.0	4.0	4.3	5.5	5.0	
Mean	4.7±2.2	4.7±2.3	4.1±2.0	4.3±2.1	4.9±1.9	5.1±1.7	5.3±2.3	
>6.0	59	29	4	5	3	5	13	.654
≦6.0	227	111	19	34	14	13	36	
Data missing	2	1	0	1	0	0	0	
**LNR (**≧**0.18)**								
Range	0.00–1.00	0.00–1.00	0.03–1.00	0.00–0.50	0.04–0.50	0.03–0.85	0.04–0.71	
Median	0.15	0.18	0.13	0.09	0.15	0.18	0.13	
Mean	0.20±0.18	0.22±0.18	0.17±0.20	0.16±0.15	0.22±0.16	0.24±0.23	0.19±0.16	
≧0.18	126	71	7	14	8	9	17	.193
<0.18	162	70	16	26	9	9	32	
**TNM stage**								
IIIA	26	11	4	5	0	2	4	.253
IIIB	188	89	19	26	12	11	31	
IIIC	74	41	0	9	5	5	14	
**ECOG**								
>2	29	1	1	0	0	4	23	**<.001**
≦2	259	140	22	40	17	14	26	
**Time interval from the operation to the start of adjuvant therapy (months)**								
Range	0.0–7.2	0.4–5.4	0.7–3.6	0.6–2.2	0.5–4.2	0.5–4.1	0.0–7.2	
Median	1.0	1.0	0.9	1.0	0.93	0.9	1.1	
Mean	1.2±0.8	1.1±0.6	1.1±0.6	1.1±0.4	1.1±0.8	1.2±0.9	1.6±1.5	
>2 Mo	24	7	1	2	1	3	10	**.014**
≦2 Mo	264	134	22	38	16	15	39	
**Adding cycles of adjuvant therapy (>2months)**								
Yes	73	11	12	9	2	10	29	**<.001**
No	215	130	11	31	15	8	20	
**Disease Status**								
Death	47	15	2	4	8	2	16	
Alive with disease	47	26	4	9	1	2	5	
Alive w/o disease	194	100	17	27	8	14	28	
**Follow-up (months)**								
Range	2.1–92.6	7.6–92.6	9.6–60.0	6.3–69.8	13.1–92.5	13.6–61.2	2.1–90.4	
Median	43.5	43.5	40.7	45.7	76.8	44.6	39.1	
Mean	43.7±17.9	43.4±14.7	37.5±15.0	44.1±12.7	68.8±26.7	41.4±14.6	39.2±21.8	

As shown in Table 1, patients with old age (ρ = 0.264, *P*<0.001) and poor performance status (ρ = 0.400, *P*<0.001) were highly selected in UFUR therapeutic group. The duration of chemotherapy was longer in the oral chemotherapy group than the others and the similar phenomenon was observed in XELODA, UFUR groups (ρ = 0.341, *P*<0.001). Except to above three factors, there were no significant difference in variables among all therapeutic groups.

### Survival in relation to all patients’ characteristics

**Fig 1:**
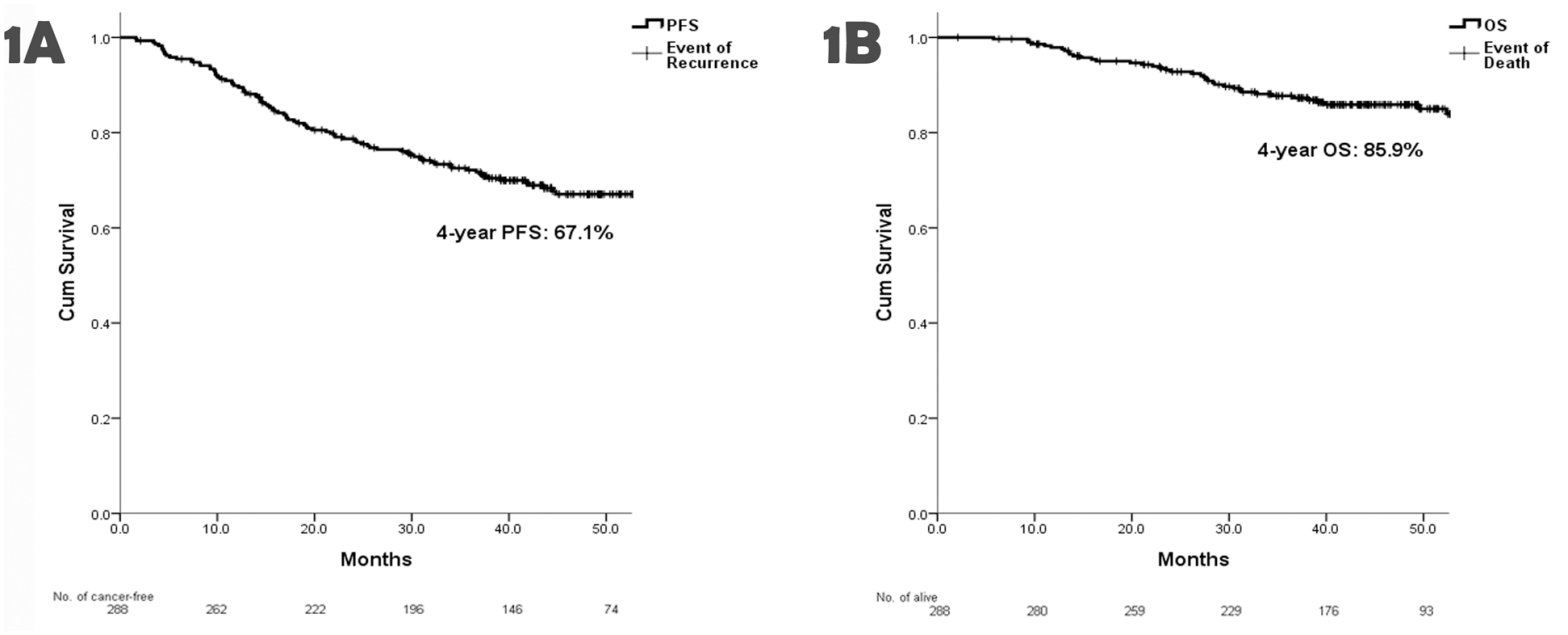
The 4-year PFS and OS. (A) The 4-year PFS was 67.1% and (B) the 4-year OS was 85.9% in all patients.

All clinic-pathologic and therapeutic factors were examined to determine their associations with progression-free survival (PFS) and OS (Table 2). The 4-year PFS and OS of 288 patients were 67.1% and 85.9% respectively (Fig 1A and 1B). The 4-year PFS and OS were significantly higher in patients aged under 70 years (*P* = 0.019 and 0.007, respectively). The PFS was higher in females than in males (*P* = 0.008) and difference in OS between genders was insignificant (*P* = 0.074). The significantly high PFS and OS rates were also noted in the patients with low LNR (<0.18) (*P*<0.001 and = 0.005, respectively) and good performance status (ECOG ≦2) (*P* = 0.027 and <0.001, respectively). The PFS of the patients with normal CEA level before operation was statistically high (*P* = 0.002). For TNM staging, compared with the patients of staged IIIC, those of stage IIIB had significantly high PFS [relative risk (RR) 0.404, 95% confidence interval (CI) 0.264–0.618, *P*<0.001] and OS (RR 0.492, 95% CI 0.273–0.887, *P* = 0.018). There was no significantly difference in survival rates among other clinic-pathologic factors.

**Table 2:** Univariate Cox regression analysis of 4-year progression-free survival and overall survival in relation to patients’ characteristics. Abbreviations: 4-y OS: 4-year overall survival; 4-y PFS: 4-year progression-free survival; 95% CI: 95% confidence interval; CEA: carcinoembryonic antigen; ECOG: Eastern Cooperative Oncology Group performance status; FL: 5-Fluorouracil (5-FU)/leucovorin; FOLFOX: mFOLFOX 6, 5-FU/leucovorin/oxaliplatin; LNR: the positive lymph node ratio of dissected lymph nodes; OXA: oxaliplatin; OXA then 5-FU: Oxaliplatin-based chemotherapy (less than 8 cycles of 5-FU/leucovorin/oxaliplatin or less than 6 cycles of oral capecitabine/intravenous oxaliplatin) followed by 5-FU-based chemotherapy (5-FU/leucovorin, oral tegafur-uracil/folinate calcium or oral capecitabine); P: probability value; RR: relative risk; TNM stage: Cancer staging system which was developed and is maintained by the American Joint Committee on Cancer (AJCC) and the Union for International Cancer Control (UICC) is based on the extent of the tumor (T), the extent of spread to the lymph nodes (N), and the presence of metastasis (M); UFUR: oral UFUR/LV, tegafur-uracil/oral folinate calcium (leucovorin); vs: versus; XELODA: oral capecitabine; XELOX: CapeOx, oral capecitabine/intravenous oxaliplatin; y/o: years old.

Patients’ Characteristics	Progression-Free Survival			Overall Survival		
	4-y PFS (%)	*P*	RR (95% CI)	4-y OS (%)	*P*	RR (95% CI)
**All patients**	67.1			85.9		
**Age (≧70)(y/o)**						
≧70	60.7	**.019**	1.642 (1.085–2.485)	77.4	**.007**	2.224 (1.244–3.977)
<70	70.9			90.5		
**Sex (Male)**						
Male	59.5	**.008**	1.793 (1.166–2.756)	82.4	.074	1.729 (0.948–3.154)
Female	75.2			89.7		
**Tumor Site (R-colon)**						
R-colon	65.6	.969	1.008 (0.664–1.532)	83.0	.342	1.321 (0.744–2.344)
L-colon	68.0			87.8		
**Pre-operative CEA (ng/ml)**						
>5.00	53.5	**.002**	1.954 (1.269–3.008)	82.2	.231	1.474 (0.781–2.781)
≦5.00	71.9			87.2		
**Histological Grade**		.532			.192	
Well	37.5	.525	1.626 (0.323–7.280)	66.7	.701	1.506 (0.187–12.147)
Moderate	67.9	.518	0.818 (0.445–1.505)	87.2	.114	0.537 (0.249–1.161)
Poor	61.3			77.1		
**Tumor Size (>6)(cm)**						
>6.0	62.3	.194	1.374 (0.850–2.222)	77.0	.078	1.762 (0.939–3.306)
≦6.0	68.0			87.9		
**LNR (≧0.18)**						
≧0.18	54.4	**<.001**	2.108 (1.387–3.204)	77.5	**.005**	2.349 (1.294–4.261)
<0.18	77.3			92.4		
**TNM stage**		**<.001**			**.023**	
IIIA	80.4	**.014**	0.312 (0.123–0.792)	96.2	.075	0.161 (0.021–1.204)
IIIB	74.1	**<.001**	0.404 (0.264–0.618)	87.9	**.018**	0.492 (0.273–0.887)
IIIC	45.9			77.3		
**ECOG (>2)**						
>2	56.9	**.027**	1.946 (1.079–3.511)	55.5	**<.001**	4.654 (2.435–8.896)
≦2	68.2			89.0		
**Time interval from the operation to the start of adjuvant therapy (months)**						
>2 Mo	66.2	.990	0.995 (0.460–2.154)	80.9	.108	2.029 (0.855–4.813)
≦2 Mo	67.1			86.3		
**Adding cycles of adjuvant therapy (>2months) (Yes)**						
Yes	68.2	.933	1.020 (0.639–1.630)	75.9	**.030**	1.928 (1.065–3.489)
No	66.5			89.1		
**Chemoregimens (vs FOLFOX)**		.881			**.016**	
FOLFOX	69.1			88.5		
XELOX	72.7	.982	0.990 (0.420–2.334)	92.3	.992	1.008 (0.230–4.414)
OXA then 5-FU	61.2	.348	1.327 (0.734–2.398)	92.3	.958	0.971 (0.322–2.929)
FL	64.7	.788	1.117 (0.499–2.500)	82.4	.089	2.248 (0.884–5.712)
XELODA	76.9	.602	0.761 (0.272–2.124)	87.7	.840	1.164 (0.266–5.097)
UFUR	63.0	.447	1.246 (0.707–2.196)	69.8	**.001**	3.396 (1.668–6.913)
**Chemoregimens (OXA-based vs 5-FU-based)**						
OXA-based	67.1	.852	1.045 (0.659–1.655)	88.7	**.009**	0.450 (0.248–0.817)
5-FU-based	67.5			78.7		
**Chemoregimens (only oral regimens vs IV or combined regimens)**						
only oral regimens^a^	69.2	.797	0.936 (0.564–1.552)	77.4	**.023**	2.021 (1.101–3.711)
combined regimens^b^	66.4			88.2		

^a^. only oral regimens: XELODA and UFUR groups;

^b^. combined regimens: FOLFOX, XELOX, OXA then 5-FU and FL groups.

Regarding therapeutic factors, adding cycles of adjuvant therapy had significant poor OS (*P* = 0.030). For regimens, the OS of the UFUR group was statistically worse than that of the FOLFOX group (RR 3.396, 95% CI 1.668–6.913, *P* = 0.001). In addition, the patients under oxaliplatin-based chemotherapies had significantly better OS than those under only 5-FU-based regimens (*P* = 0.009), but the patients under only oral regimens had better OS than those under IV or combined regimens (*P* = 0.023).

### Impact of age in relation to therapeutic strategies

In the analysis of our patients’ distribution according to chemoregimens shown in Table 1, the patients aged under 70 received more intensive therapies than those aged over 70 (*P*<0.001). Hence, we did advanced analysis in therapeutic factors between the patients aged under 70 (180 patients) and those over 70 (108 patients) (Tables 3 and 4).

**Table 3:** Univariate Cox regression analysis of 4-year progression-free survival and overall survival of 180 patients with age <70 y/o in relation to chemotherapeutic strategies. Abbreviations: 4-y OS: 4-year overall survival; 4-y PFS: 4-year progression-free survival; 95% CI: 95% confidence interval; FL: 5-Fluorouracil (5-FU)/leucovorin; FOLFOX: mFOLFOX 6, 5-FU/leucovorin/oxaliplatin; LNR: the positive lymph node ratio of dissected lymph nodes; OXA: oxaliplatin; OXA then 5-FU: Oxaliplatin-based chemotherapy (less than 8 cycles of 5-FU/leucovorin/oxaliplatin or less than 6 cycles of oral capecitabine/intravenous oxaliplatin) followed by 5-FU-based chemotherapy (5-FU/leucovorin, oral tegafur-uracil/folinate calcium or oral capecitabine); P: probability value; RR: relative risk; UFUR: oral UFUR/LV, tegafur-uracil/oral folinate calcium (leucovorin); vs: versus; XELODA: oral capecitabine; XELOX: CapeOx, oral capecitabine/intravenous oxaliplatin; y/o: years old.

Patients’ Characteristics	Progression-Free Survival			Overall Survival		
	4-y PFS (%)	*P*	RR (95% CI)	4-y OS (%)	*P*	RR (95% CI)
**Chemoregimens (vs FOLFOX)**		.923			.296	
FOLFOX	70.9			89.8		
XELOX	86.7	.374	0.521 (0.124–2.189)	100.0	.897	0.873 (0.111–6.843)
OXA then 5-FU	68.4	.831	1.085 (0.512–2.300)	100.0	.965	0.000 (0.000–0.000)
FL	69.2	.761	1.155 (0.456–2.926)	92.3	.221	2.016 (0.657–6.188)
XELODA	85.7	.540	0.536 (0.073–3.942)	85.7	.595	1.748 (0.223–13.712)
UFUR	64.3	.911	1.063 (0.368–3.068)	65.6	**.017**	3.780 (1.263–11.315)
**Chemoregimens (OXA-based vs 5-FU-based)**						
OXA-based	71.2	.829	1.084 (0.522–2.251)	92.1	**.037**	0.385 (0.157–0.946)
5-FU-based	72.1			83.4		
**Chemoregimens (only oral regimens vs IV or combined regimens)**						
only oral regimens^a^	75.4	.529	0.720 (0.258–2.007)	76.6	.077	2.471 (0.906–6.736)
combined regimens^b^	70.3			92.1		
**Adding cycles of adjuvant therapy (>2months) (Yes)**						
Yes	75.8	.579	0.814 (0.393–1.684)	81.4	.052	2.359 (0.991–5.617)
No	69.6			92.7		

^a^. only oral regimens: XELODA and UFUR groups;

^b^. combined regimens: FOLFOX, XELOX, OXA then 5-FU and FL groups.

**Table 4:** Univariate Cox regression analysis of 4-year progression-free survival and overall survival of 108 patients with with old age (≧70) in relation to chemotherapeutic strategies. Abbreviations: 4-y OS: 4-year overall survival; 4-y PFS: 4-year progression-free survival; 95% CI: 95% confidence interval; FL: 5-Fluorouracil (5-FU)/leucovorin; FOLFOX: mFOLFOX 6, 5-FU/leucovorin/oxaliplatin; LNR: the positive lymph node ratio of dissected lymph nodes; OXA: oxaliplatin; OXA then 5-FU: Oxaliplatin-based chemotherapy (less than 8 cycles of 5-FU/leucovorin/oxaliplatin or less than 6 cycles of oral capecitabine/intravenous oxaliplatin) followed by 5-FU-based chemotherapy (5-FU/leucovorin, oral tegafur-uracil/folinate calcium or oral capecitabine); P: probability value; RR: relative risk; UFUR: oral UFUR/LV, tegafur-uracil/oral folinate calcium (leucovorin); vs: versus; XELODA: oral capecitabine; XELOX: CapeOx, oral capecitabine/intravenous oxaliplatin; y/o: years old.

Patients’ Characteristics	Progression-Free Survival			Overall Survival		
	4-y PFS (%)	*P*	RR (95% CI)	4-y OS (%)	*P*	RR (95% CI)
**Chemoregimens (vs FOLFOX)**		.548			.328	
FOLFOX	64.9			85.0		
XELOX	46.9	.355	1.700 (0.552–5.236)	75.0	.909	1.133 (0.132–9.707)
OXA then 5-FU	40.0	.120	2.160 (0.819–5.695)	70.0	.078	3.269 (0.876–12.195)
FL	50.0	.823	1.196 (0.249–5.739)	50.0	.199	3.159 (0.545–18.310)
XELODA	71.6	.622	0.729 (0.208–2.561)	88.9	.748	0.703 (0.082–6.020)
UFUR	61.9	.948	1.026 (0.475–2.216)	71.5	.078	2.598 (0.897–7.519)
**Chemoregimens (OXA-based vs 5-FU-based)**						
OXA-based	56.9	.277	1.415 (0.757–2.646)	79.2	.488	0.748 (0.329–1.698)
5-FU-based	65.0			75.5		
**Chemoregimens (only oral regimens vs IV or combined regimens)**						
only oral regimens^a^	65.9	.259	0.694 (0.367–1.310)	77.8	.684	1.182 (0.529–2.641)
combined regimens^b^	56.5			77.3		
**Adding cycles of adjuvant therapy (>2months) (Yes)**						
Yes	60.8	.930	0.972 (0.510–1.850)	69.4	.522	1.305 (0.578–2.946)
No	60.0			81.3		

^a^. only oral regimens: XELODA and UFUR groups;

^b^. combined regimens: FOLFOX, XELOX, OXA then 5-FU and FL groups.

**Fig 2:**
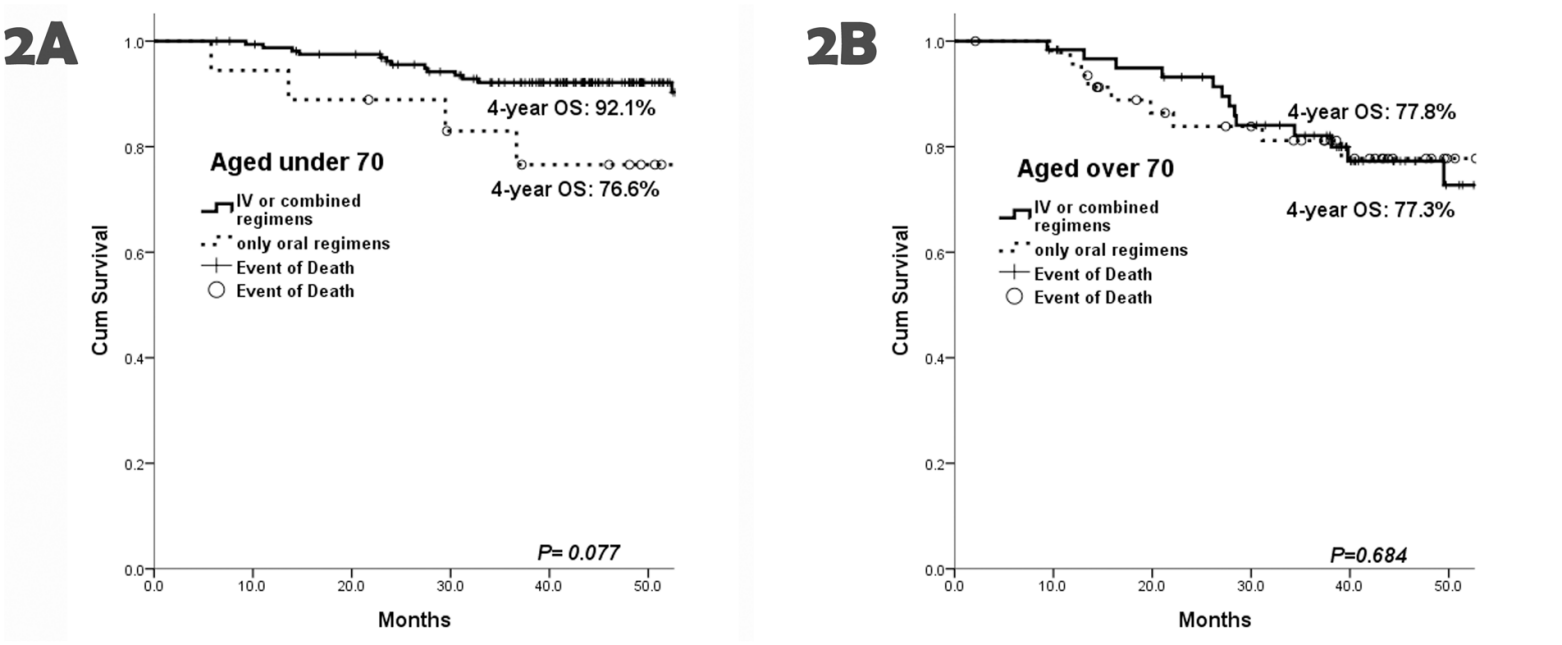
The 4-year OS between the patients with combined therapies and those with only oral chemotherapies. (A) Markedly higher 4-year OS was noted in the patients aged under 70 with combined therapies than that in these patients with only oral chemoregimens (4-year OS: 92.1% vs 76.6%, respectively, *P* = 0.077). In contrast to the separated survival curves in the patients aged under 70, (B) the curves were crossed between combined therapies and only oral chemotherapies in the old patients (4-year OS: 77.3% vs 77.8%, *P* = 0.684).

**Fig 3:**
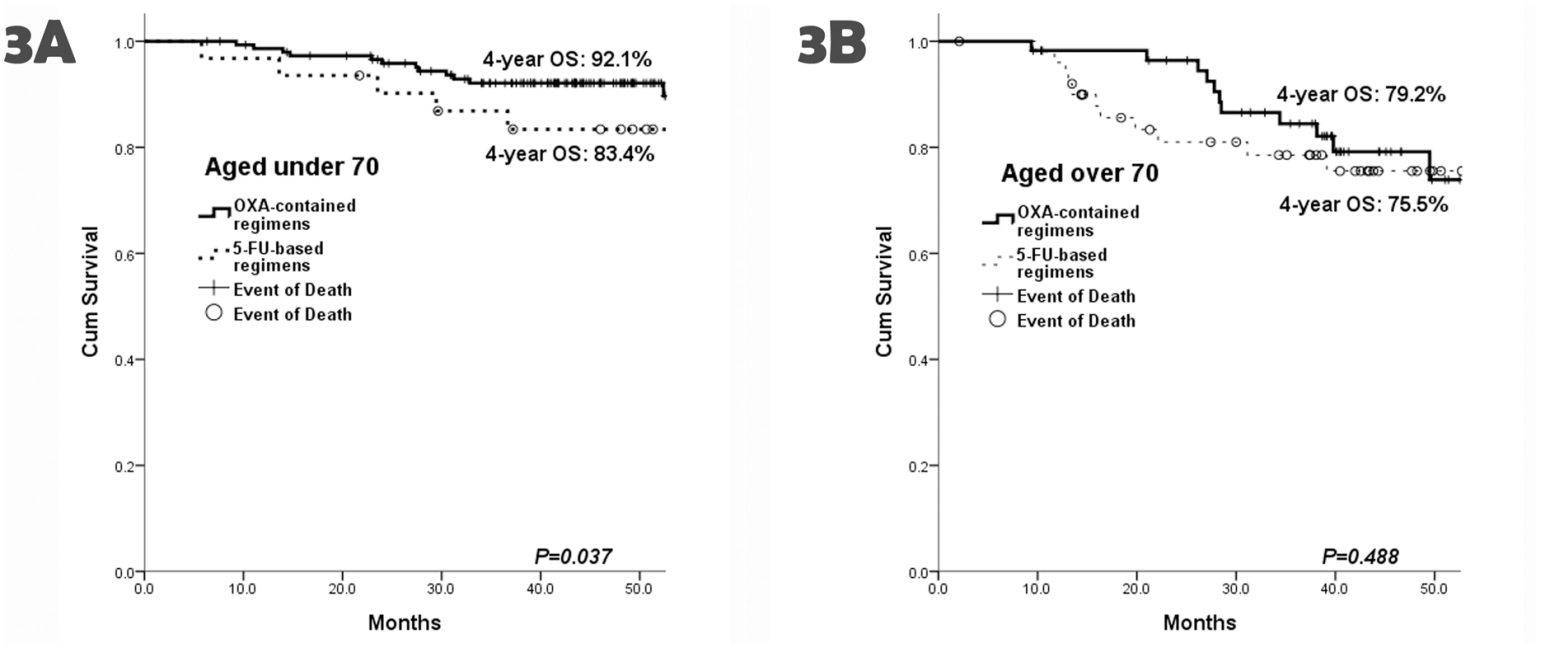
The 4-year OS between the patients with oxaliplatin-contained regimens and those with 5-FU-based therapies. (A) For the patients aged under 70, significant differences in 4-year OS were noted between they received oxaliplatin-contained regimens and 5-FU-based therapies (92.1% vs 83.4%, respectively, RR 0.385, 95% CI 0.157–0.946, *P* = 0.037). (B) There was no significantly survival benefit in the old patients as they received oxaliplatin-contained therapies (79.2% vs 75.5%, *P* = 0.488).

For the patients aged under 70, relatively low 4-year PFS and OS were noted in UFUR group (4-year PFS 64.3% and 4-year OS 65.6%), but there was only significant difference in OS between UFUR group and FOLFOX group (RR 3.780, 95% CI 1.263–11.315, *P* = 0.017). In addition, markedly high 4-year OS were also noted in the patients with intensive IV or combined therapy (not reach statistical significance) (4-year OS: 92.1% vs 76.6%, with only oral chemotherapy, *P* = 0.077) (Fig 2A) or even in those with oxaliplatin-contained regimens (4-year OS: 92.1% vs 83.4%, with only 5-FU-based regimens, *P* = 0.037) (Fig 3A).

Additionally, there was no significant difference in survival rates among all therapeutic groups. In contrast, patients aged under 70 exhibited higher OS rates. There was no significant benefit for survival in the old age group as they received aggressive IV or combined therapy (4-year OS: 77.3% vs 77.8%, only oral chemotherapy, *P* = 0.684) (Fig 2B). The similar finding was also found between oxaliplatin-contained therapies and non-oxaliplatin-contained regimens for the old patients (4-year OS: 79.2% vs 75.5%, *P* = 0.488) (Fig 3B).

Furthermore, prolonged cycles of adjuvant therapy (additional treatment>2 months) did not provide significantly survival benefit regardless of ages.

## Discussion

**Fig 4:**
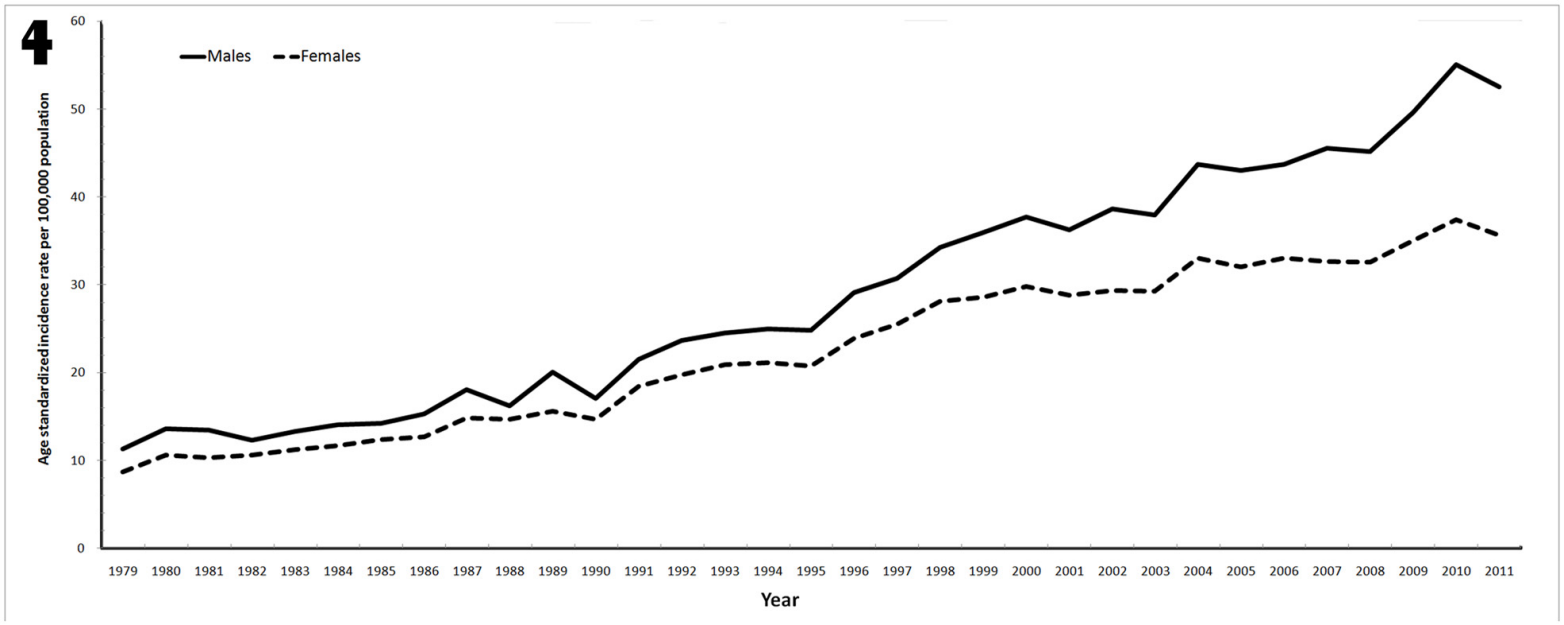
Long-term trends in age-standardized incidence of colorectal cancer in Taiwan between 1979 and 2011. The prevalence of colorectal cancer has been increased gradually in the past 30 years. [Adapted from Taiwan Cancer Registry. (2014, March 19). Age-standardized incidence of long-term trends- digestive organs and peritoneum.].

In Taiwan, the prevalence of colorectal cancer has been increased gradually in the past 30 years [23] (Fig 4). As most patients are diagnosed in an advanced stage, selection of therapeutic strategy remains a tremendous challenge for oncologists, especially for stage III colon cancer. In the present study, we retrospectively examined patterns of adjuvant chemotherapy used in patients with stage III colon cancer based on patients’ information from Cancer Registry Group at Tri-Service General Hospital incorporating with medical chart review between January 2006 and October 2014. During designated time period, oxaliplatin has been used intensively as adjuvant therapy and become the principal chemotherapeutic agent in the adjuvant regimen. However, increasing patient age and diminished performance status have been still inversely associated with the addition of oxaliplatin to adjuvant chemotherapy regimens. Alternative treatment strategy based on oral chemotherapy is worth discussing.

In this study, our results show that oral chemotherapy was preferably chosen for patients with old age (44.4%) or poor performance status (93.1%). In the past, choice of chemotherapeutic regimen for aforementioned patients has been influenced and restricted due to toxicity profile of IV regimens or suboptimal care. Interestingly, analyzing associated clinic-pathologic factors, it is found that old age and poor performance status actually played the important roles. However, 93.1% patients with poor performance status in this study underwent oral chemotherapy and the selection bias resulted in failure of analysis among all therapeutic groups. We, therefore, focused on the difference in all therapeutic groups between the patients aged under 70 and those over 70.

### Oxaliplatin-contained therapy

For the old age patients, our data showed that all therapeutic factors were insignificant. There was no statistical significance in oxaliplatin-contained therapy and close survival rates were noted between the patients undergoing oxaliplatin-contained therapy and those with only 5-FU-based regimens. In contrast to the patients aged under 70, the survival rates were significantly higher for patients prescribed with oxaliplatin, especially in 4-year OS (oxaliplatin-contained vs only 5-FU-based: 92.1% vs 83.4%, RR 0.385, 95% CI 0.157–0.946, *P* = 0.037). McCleary NJ et al. analyzed two of the oxaliplatin-based trials {Multicenter International Study of Oxaliplatin/Fluorouracil/Leucovorin in the Adjuvant Treatment of Colon Cancer (MOSAIC) [6] and National Surgical Adjuvant Breast and Bowel Project (NSABP)-C07 [7]} and found statistically significantly improved survival for patients with oxalipatin-contained regimens among patients aged under 70 but not those over 70 [24]. In consistent with their findings, our results suggested of non-benefit from oxaliplatin in the adjuvant setting for old-age patients.

A recent study by McCleary NJ et al. has reported that there was no statistically significant difference between patients and controls for combined endpoints of any grade≧3 toxicity or hospital/facility admission based on the data of 84 patients (advanced stage, including metastatic disease) aged more than 75 years undergoing oxaliplatin-contained chemotherapy regimens [25]. It is suggested that safety and efficacy of oxaliplatin-contained chemotherapy regimens are not associated with patient age. Hence, use of oxaliplatin in the adjuvant setting for patients with staged III disease and old age requires a re-evaluation for the association of age with tolerance or toxicity.

### Oral chemotherapy

Two trials {X-ACT (Xeloda in Adjuvant Colon Cancer Therapy) [4] and NSABP-C06 [17]} were designed to demonstrate non-inferiority of oral fluoropyrimidine therapy compared with IV FL. McCleary NJ et al. re-analyzed the result based on the variable of age and found that there was no statistical significance for survival between treatment and age [26]. It is suggested that the therapeutic benefit of oral fluoropyrimidines is comparable to that of IV FL regardless of age. In our study, there was also no statistical significance for survival rates in selecting oral or IV, combined chemotherapies in the patients aged over 70. Taking consideration of convenience, medication compliance and non-benefit from oxaliplatin for old patients into account, oral fluoropyrimidines represent an ideal candidate with preferable efficacy. On the other hand, for the patients aged under 70, markedly lower 4-year OS (76.6% vs 92.1%, IV or combined therapy) was present as they only received oral chemotherapies though the difference did not reach statistical significance (*P* = 0.077). The finding highlights the influence from oxaliplatin-contained therapy [161 patients received IV or combined therapy, of whom there were 148 (91.9%) patients underwent oxaliplatin-contained strategies] and implies that benefit of oxaliplatin-contained therapy is restricted to patients aged under 70 for OS.

### Clinic-pathologic factors

In our study, marked differences were found in PFS for several variables including gender and CEA level. It has been reported that that preoperative serum concentration of CEA was an independent prognostic factor in patients with stage II or III colon cancer [27]. However, opponents of CEA testing argue that approximately 40% of all colorectal recurrences are not associated with increased CEA levels [28]. Although CEA is widely used as diagnostic and prognostic factor, its utility remains controversial [29].

Significantly lower PFS was found in males than that in females in our study. Colonoscopic screening of asymptomatic individuals has corroborated male sex as a risk factor for the development of colon cancer in all age groups [30,31]; however, whether this disparity depends on protective factors in women, tumor-promoting factors in males, or both is unknown. A protective role of female hormones against the development of frank colorectal cancer is suggested by data from the Women’s Health Initiative (WHI). In the WHI, two large randomized controlled trials examined the effects of hormonal replacement therapy on postmenopausal women over a 5-year period, using colorectal cancer development as one of the endpoints. The first study showed that combined treatment with both equine estrogen (E2) and medroxyprogesterone acetate (MPA) substantially reduced the risk of colorectal cancer compared with placebo (odds ratio, 0.63) after a 5-year follow-up [32]. However, protection was not found in a second randomized controlled trial among women who had previously undergone hysterectomy and were treated solely with equine estrogen (odds ratio, 1.08) [33]. Although treatment with a combination of female hormones may be protective against a 5-year incidence of colorectal cancer in postmenopausal women, whether this effect involves the same mechanism as that in the differences between the sexes in adenomagenesis and colorectal cancer is still unknown.

Sukamal Saha et al. addressed predicting long-term survival in colon cancer patients by tumor size from analysis of National Cancer Data Base (NCDB) [19]. Their results showed that patients with tumor size of 4–6cm and > 6cm exhibited a 23% and 70% increased risk of death over 5-years, respectively. Our data showed no significance of poor PFS or OS in our patients with tumor size > 6cm. This result may suggest that more well-designed studies in investing the role of primary tumor size in colon cancer prognosis are necessary.

In general, outcome prediction based on tumor stage using the AJCC TNM system is currently considered as the strongest prognostic indicator for patients with colon cancer [34]. In our study, there were few patients staged IIIA (9.0%) and the uneven distribution might lead to less significance in assessment of OS (*P* = 0.075) between staged IIIA and IIIC. In this study, hence, the survival curves were observed to cross. Otherwise, in our study, the new marker of LNR reached statistical significance in predicting poor PFS and OS. The finding suggested that LNR seem to be reliable and may provide additional information when used in conjunction with the AJCC TNM system.

### Other therapeutic factors

Delayed adjuvant therapy and duration of therapy or prolonged therapeutic course are worth a discussion for stage III colon cancer. Hershman D et al. reported that delayed initiation of adjuvant therapy was associated with both cancer-specific and all-cause mortality in old patients with stage III colon cancer [35]. Later, several meta-analyses have confirmed that delayed administration of adjuvant chemotherapy after curative surgery is associated with significantly low overall survival [36,37]. In our study, there were only 24 (8.3%) patients with delayed adjuvant chemotherapy (initiation of adjuvant therapy for more than 2 months after surgery). For survival analysis, there was less statistical significance; however, marked lower survival rates were still noted in the patients with delayed therapy.

With regards to therapeutic duration, in this study, there were 73 (25.3%) patients with prolonged treatment more than 2 months and no benefit was found from this therapeutic setting. A recent meta-analysis also confirmed that adjuvant chemotherapy of colorectal cancer should not last for more than 6 months and prolonged duration would result in lower benefit to risk ratio [38]. Hence, 6-month adjuvant therapy may be the adequate strategy for these patients.

### The limitation of our study

The retrospective study was conducted at a single institution with small patient numbers and there were some biases on study design. First, we did not include any molecular markers of colon cancer, including microsatellite instability (MSI) status. Although use of molecular profiling of colon cancer in the clinical practice is promising, it has not been considered as routine examination in cancer management. Due to lack of molecular information, we only examined clinic-pathologic characteristics, therapeutic factors and assessed the practice settings. In the future, integration of molecular characteristics may have potential to accurately predict the outcome of stage III colon cancer. Second, for the issue of cancer in elder patients, we did not take the side effects of various therapies and the quality of life into account. The quality of life is an outcome that is rarely measured in the past studies [39]. In additions, many confounding factors will be present in the analysis of quality of life, such as the patients’ underlying co-morbidities, intensity of the original therapeutic strategy…etc. Hence, we did not discuss side-effect and quality of life on our study. Instead of this problem, survival is of clear clinical relevance and we used OS as the end-point of our study. However, for cancer in elder patients, the quality of life is still an important issue and further studies with the useful quality of life index are encouraged. Finally, the population of our study was still too small and the findings were limited. However, the significant findings in selecting chemotherapeutic strategies can still provide the effective options for the patients and the clinicians. The results suggest that benefit of oxaliplatin-contained therapy is restricted to patients aged under 70 and only oral fluoropyrimidines may be an effective option for old patients. Moreover, the clinic-pathologic factors, including LNR and TN stage, seem to have influence on outcome of stage III colon cancer. These findings indicate good directions for development of appropriate clinic predictors for colon cancer patients under adjuvant therapy.

## Conclusion

Poor prognosis of stage III colon cancer has been a concern for oncologists, leading to development of potential therapeutic strategies. Many predictive factors such as molecular markers are also evaluated for these patients in recent. However, the adequate and effective individualized therapeutic strategy plays an important role in cancer treatment. The findings of present study highlight that the therapeutic strategies shall be individualized for the different age groups. Moreover, the results suggest that a great consideration shall be made for selection of treatments and choice of chemotherapeutics in cancer patients. Prospectively, well-designed studies encompassing a larger patient population are necessary to elucidate the efficacy of this therapeutic setting and the results shall contribute to a development of adequate, tolerable treatments for colon cancer patients.
